# Highly Modular Protein Micropatterning Sheds Light on the Role of Clathrin-Mediated Endocytosis for the Quantitative Analysis of Protein-Protein Interactions in Live Cells

**DOI:** 10.3390/biom10040540

**Published:** 2020-04-02

**Authors:** Peter Lanzerstorfer, Ulrike Müller, Klavdiya Gordiyenko, Julian Weghuber, Christof M. Niemeyer

**Affiliations:** 1 University of Applied Sciences Upper Austria, 4600 Wels, Austria; ulrike.mueller@fh-wels.at; 2 Karlsruhe Institute of Technology (KIT), Institute for Biological Interfaces (IBG-1), 76344 Eggenstein-Leopoldshafen, Germany; klavdiya.gordiyenko@kit.edu

**Keywords:** micropatterning, protein-protein interactions, clathrin-mediated endocytosis, fluorescence microscopy, DNA origami

## Abstract

Protein micropatterning is a powerful tool for spatial arrangement of transmembrane and intracellular proteins in living cells. The restriction of one interaction partner (the bait, e.g., the receptor) in regular micropatterns within the plasma membrane and the monitoring of the lateral distribution of the bait’s interaction partner (the prey, e.g., the cytosolic downstream molecule) enables the in-depth examination of protein-protein interactions in a live cell context. This study reports on potential pitfalls and difficulties in data interpretation based on the enrichment of clathrin, which is a protein essential for clathrin-mediated receptor endocytosis. Using a highly modular micropatterning approach based on large-area micro-contact printing and streptavidin-biotin-mediated surface functionalization, clathrin was found to form internalization hotspots within the patterned areas, which, potentially, leads to unspecific bait/prey protein co-recruitment. We discuss the consequences of clathrin-coated pit formation on the quantitative analysis of relevant protein-protein interactions, describe controls and strategies to prevent the misinterpretation of data, and show that the use of DNA-based linker systems can lead to the improvement of the technical platform.

## 1. Introduction

Simple and robust quantitation of protein-protein interactions (PPIs) in living cells remains challenging. Widespread approaches are mainly based on biochemical and genetic methods such as co-immunoprecipitation [1], pull-down experiments [2], affinity purification [3], and yeast two-hybrid screens [4]. On the other hand, current biophysical techniques for quantitative protein interaction analysis in live cells include fluorescence resonance energy transfer (FRET) [5], fluorescence lifetime imaging (FLIM) [6], bimolecular fluorescence complementation (BiFC) [7], fluorescence cross-correlation spectroscopy (FCS) [8], and single molecule techniques [9]. Most of these techniques require special equipment and are costly, laborious, and rather demanding in living cells. These methods do not allow for easy quantification or are based on the analysis of cell lysates. To overcome these limitations, we and others have used protein micropatterning on solid substrates to induce the reorganization of target proteins distributed in the plasma membrane of living cells for the investigation of distinctive PPIs [10,11,12,13,14,15,16,17,18,19,20,21].

**Figure 1:**
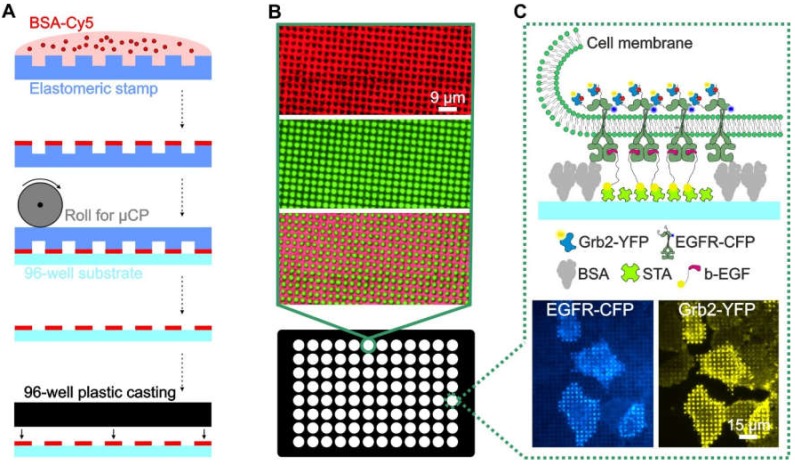
Overview and workflow of the live cell micropatterning approach. (**A**) Schematic illustration of the µCP procedure using a large-area polymeric stamp. (**B**) Total internal reflection fluorescence (TIRF) microscopy images of a single well of the ready-to-use 96-well plate. Representative images of the BSA-Cy5 grid (red, top), FITC-labeled biotinylated antibody (green, middle) and merged images (bottom) are shown. (**C**) Schematic illustration and representative images of a selected bait-prey interaction detected on the micropatterned surface. Anti-bait protein (biotinylated EGF) was arranged in regular patterns, and the bait protein (EGFR) was reorganized according to the anti-bait molecules. The prey protein (Grb2), therefore, localizes according to the bait patterns, which indicates the close proximity and/or an interaction between the bait and prey. Specific enrichment can be visualized using fluorescently tagged bait/prey molecules such as EGFR-CFP and Grb2-YFP. Abbreviations: BSA, bovine serum albumin; b-EGF, biotinylated EGF; STA, streptavidin; and µCP, microcontact printing. Illustrations are not drawn to scale.

Despite these advances, it is difficult to produce large-area patterned substrates with a modular structure that enable the systematic investigation of specific and nonspecific effects in the analysis of PPIs in adhered cells. Among the numerous methods for surface patterning of cover slips and microscopy slides, soft lithography via microcontact printing (µCP) is one of the most convenient and widely used methods [22,23]. In this case, we describe an extension of this method to functionalize the glass bottom of 96-well plates using large-area perfluoropolyether (PFPE)-based elastomeric stamps. The resulting ready-to-use multi-well plate can be functionalized in a highly modular manner to contain arbitrary micron-scale patterns of complex protein architectures that can be used for high-throughput experimental analysis of PPIs in live cells (Figure 1).

Using this approach, we investigated the potential pitfalls and difficulties in data interpretation based on the enrichment of clathrin, which is a protein essential for clathrin-mediated receptor endocytosis. We found that clathrin forms internalization hotspots within the patterned areas, which potentially leads to unspecific protein-protein co-recruitment. The consequences of clathrin-coated pit (CCP) formation on the quantitative analysis of PPIs as well as controls and strategies to prevent misinterpretation are discussed.

## 2. Materials and Methods

### 2.1. Reagents and DNA Constructs

Bovine serum albumin (BSA), streptavidin, EGF, DMSO, Tris base, EDTA, Tween-20, D-biotin, SDS, and transferrin Alexa-647 were purchased from Sigma-Aldrich (Schnellendorf, Germany). NaCl and MgCl2 × 6 H2O were purchased from AppliChem (Darmstadt, Germany). BSA-Cy5 was obtained from Protein Mods (Madison, WI, USA). Biotinylated anti-EGFR, anti-HA, mouse-IgG, and anti-mouse-IgG (FITC) were purchased from Antibodies Online (Herford, Germany). The pYFP-N1-Grb2, mSH2-Grb2-YFP and pECFP-N1-EGFR plasmids were kindly provided by Lawrence E. Samelson, (NIH, Bethesda, MD, USA), and the pEGFP-C1-CLCA1 construct was a kind gift from Eileen M. Lafer (UTHSC, San Antonio, TX, USA).

### 2.2. Cell Culture and Transfection

All cell culture reagents were purchased from Biochrom GmbH (Berlin, Germany). HeLa cells (ATCC) were cultured in RPMI medium supplemented with 10% FBS and 1% penicillin/streptomycin and grown at 37 °C in a humidified incubator with 5% CO_2_. For transient transfection, HeLa cells were sub-cultured the day before and were then transfected with plasmids using the Lipofectamine LTX reagent (Thermo Fisher Scientific, Waltham, MA, USA), according to the manufacturer’s instructions. HeLa cells stably expressing Grb2-YFP were described previously [12].

### 2.3. Microcontact Printing

Microcontact printing was performed as described previously [16] with modifications. A field of a large-area PFPE elastomeric stamp (1 µm grid size), obtained by the EV-Group (St. Florian am Inn, Upper Austria, Austria), was cut out, and washed by flushing with ethanol (100%) and distilled water. After drying with nitrogen, the stamp was incubated in 50 mL bovine serum albumin (BSA, or BSA-Cy5) solution (1 mg/mL) for 30 min. This step was followed by washing the stamp again with phosphate-buffered saline (PBS) and distilled water. After drying with nitrogen, the stamp was placed with homogeneous pressure onto the clean epoxy-coated glass bottom of a 96-well plate and incubated overnight at 4 °C. The next day, the stamp was stripped from the glass with forceps, and the glass bottom was bonded to a 96-well plastic casting using adhesive tape (3M) and closed with an appropriate lid.

### 2.4. Design, Assembly, Purification, and Quantification of DNA Origami Nanostructures

DNA origami nanostructures (DONs) were prepared as previously described [24] with slight modifications. Two different DONs were used, each of which contained 5 biotin residues for the sequential binding of streptavidin and biotinylated EGF, that were arranged in either close (denoted as “5close”) or far (“5far”) distances on the rectangular origami surface (Figure S1). The assembly was conducted in 1x TEMg buffer (20 mM Tris base, 1 mM EDTA, 12 mM MgCl_2_ × 6 H_2_O, pH 8.0) in a total volume of 500 μL. The DONs were assembled by a stepwise temperature decrease from 75 °C to 25 °C. Then, excess staple strands were removed by PEG precipitation. The obtained pellet was resuspended in 45 μL of TENaCl buffer (20 mM Tris base, 1 mM EDTA, and 250 mM NaCl, pH 7.6) and incubated overnight at 36 °C. The concentration of the purified DONs was determined by a quantitative polymerase chain reaction (qPCR). In addition, the samples were analyzed with atomic force microscopy (AFM), as shown in Figure S1.

### 2.5. Live Cell Micropatterning

#### 2.5.1. Antibody Patterning

For the live cell experiments, a reaction chamber was incubated with 100 µL streptavidin solution (50 µg/mL) and incubated for 30 min at room temperature. After washing two times with PBS, 100 µL biotinylated antibody solution (10 µg/mL) was added for 30 min at room temperature. Finally, the incubation wells were washed twice with PBS, and cells were seeded at defined cell density (depending on cell type) for the live cell microscopy analysis. The cells were allowed to attach to the surface for at least 3–4 h prior to imaging to ensure a homogeneous cell membrane/substrate interface, which is a prerequisite for quantitative total internal reflection fluorescence (TIRF) microscopy.

#### 2.5.2. DNA Origami Patterning

DNA micropatterns were assembled on microcontact printed substrates as follows: First, the reaction chamber (wells of the multi-well plate) was incubated with 100 µL streptavidin (50 µg/mL) for 30 min at room temperature and then washed four times with TETBS-150 (20 mM Tris base, 150 mM NaCl, 5 mM EDTA, and 0.05% Tween-20 [v/v], pH 7.5), twice for 30 s each time and twice for 5 min each time. Next, 50 µL of biotin-Tr1-12 oligonucleotide (240 nM) was added and incubated at room temperature for 30 min with continuous shaking. The chambers were washed three times with biotin-TETBS (20 mM Tris base, 150 mM NaCl, 5 mM EDTA, 0.05% Tween-20 [v/v], and 800 µM D-biotin), twice for 30 s each time, and once for 20 min at room temperature, which is followed by two TETBS-150 washes for 1 min each. DON preparation was performed by mixing 100 fmol biotinylated DONs with 1.67 µL streptavidin (10 µM) with PBS-/- added to a total volume of 10 µL and incubating the mixture for 30 min while shaking at room temperature. Subsequently, 5 µL of biotin-EGF (6 µM) was added and incubated for 45 min while shaking at room temperature. Next, 20 µL of biotin-TETBS was added, and the solution was again incubated for 15 min at room temperature while shaking. Finally, 50 µL TEMg with 0.05% SDS (20 mM Tris base, 2 mM EDTA, 12.5 mM MgCl_2_ × 6 H_2_O, and 0.05% SDS [m/v], pH 7.6) was added to the mixture. For DON hybridization, 90 µL of the DON mixture was incubated on the oligonucleotide-functionalized surfaces for 2 h at room temperature. The chambers were washed 4 times with TETBS-300 (20 mM Tris base, 300 mM NaCl, 5 mM EDTA, and 0.05% Tween-20 [v/v], pH 7.5), twice for 30 s and twice for 5 min, which is followed by 2 washes with PBS-/-for 1 min each time. Subsequently, the cells were seeded and incubated at 37 °C for approximately 3 h to allow for homogeneous cell attachment to the prepared surface, which is followed by live cell microscopy analysis via TIRF microscopy.

### 2.6. Live Cell TIRF Microscopy

The detection system was set up on an epi-fluorescence microscope (Nikon Eclipse Ti2). A multi-laser engine (Toptica Photonics, Munich, Germany) was used for selective fluorescence excitation of CFP, GFP, YFP, RFP, and Alexa-647/Cy5 at 405, 488, 516, 561, and 640 nm, respectively. The samples were illuminated in total internal reflection (TIR) configuration (Nikon Ti-LAPP) using a 60x oil immersion objective (NA = 1.49, APON 60XO TIRF). After appropriate filtering using standard filter sets, the fluorescence was imaged onto a sCMOS camera (Zyla 4.2, Andor, Northern Ireland). The samples were mounted on an x-y-stage (CMR-STG-MHIX2-motorized table, Märzhäuser, Germany), and scanning of the larger areas was supported by a laser-guided automated Perfect Focus System (Nikon PFS).

### 2.7. Contrast Quantitation and Statistical Analysis

Contrast analysis was performed as described previously [12]. In short, initial imaging recording was supported by the Nikon NIS Elements software. Images were exported as TIFF frames and fluorescence contrast analysis was performed using the Spotty framework [25]. The fluorescence contrast <c> was calculated as <c> = (F^+^ - F^−^)/(F^+^ - F_bg_), where F^+^ denotes the intensity of the inner pixels of the pattern. F^−^ shows the intensity of the surrounding pixels of the micropattern, and F_bg_ shows the intensity of the global background. Data are expressed as the means ± SE. An unpaired *t*-test was used to compare two experimental groups. Comparisons of more than two different groups were performed using two-way ANOVA, which was followed by Tukey’s multiple comparisons test in GraphPad Prism software (version 7).

## 3. Results and Discussion

### 3.1. Large-Area µCP for High-Throughput Micropatterning Experiments

As shown in Figure 1A, the protein patterned cell substrate was produced by printing a BSA (for surface passivation) grid on an epoxysilane-coated glass surface, where the resulting unblocked 1 µm patterns were filled with streptavidin, to which biotinylated antibodies or ligands could be attached (Figure 1B). The immobilized antibody/ligand can bind to the extracellular domain of the membrane protein of interest, herein referred to as the “bait protein”, for instance, a cell surface receptor. The bait protein will reorganize in the plasma membrane, according to the printed regular anti-bait antibody/ligand patterns. This enables the investigation of bait-dependent PPIs, as the redistribution to the bait-enriched areas of a putative interaction partner (herein referred to as the “prey”) can be easily monitored using fluorescence labels. The use of streptavidin micropatterns allows for the easy integration of biotinylated DNA oligonucleotides, which enables a very high degree of modularity in the functionalization of the patterned surfaces, as discussed below.

In Figure 1C, a schematic illustration as well as representative total internal reflection fluorescence (TIRF) microscopy images of the selected bait-prey interaction between the epidermal growth factor receptor (EGFR) and the downstream molecule Grb2 are shown. Using this method, we recently investigated the downstream signaling events of different receptor families, including G protein-coupled receptors (GPCRs) such as the β_2_-adrenergic receptor [15], tyrosine kinase receptors (TKRs) such as the insulin-like growth factor (IGF), insulin receptor [16], and EGFR [12,17]. These earlier studies provided evidence that the surface-patterning approach can lead to the accumulation of proteins critical for receptor-mediated endocytosis inside the bait-enriched areas that might directly affect bait/prey copatterning and the subsequent quantitative analysis.

### 3.2. Protein Patterning Affects Clathrin Distribution in Live Cells

In a previous study [12], we observed that clathrin, which plays a major role in the formation of coated vesicles and, therefore, receptor internalization [26], can be densely clustered in EGFR-enriched micropatterns. The formation of these internalization hotspots might lead to misinterpretation of bait-prey interactions as well as for the detection of false-positive bait/prey colocalization events, when the investigated receptors are desensitized via clathrin-mediated endocytosis (CME). To further elaborate on this issue, we used our experimental high-throughput platform to investigate whether protein micropatterning itself might lead to clathrin assembly within the patterned areas. To this end, we used EGFR (as bait) with the adapter protein Grb2 (as prey) for a more detailed analysis of bait/prey recruitment. Grb2 was frequently reported to directly bind to phosphorylated tyrosine-containing peptides on receptors (such as EGFR) via its SH2 domain, which results in the activation of downstream kinases as well as the induction of EGFR endocytosis [27,28,29,30].

**Figure 2:**
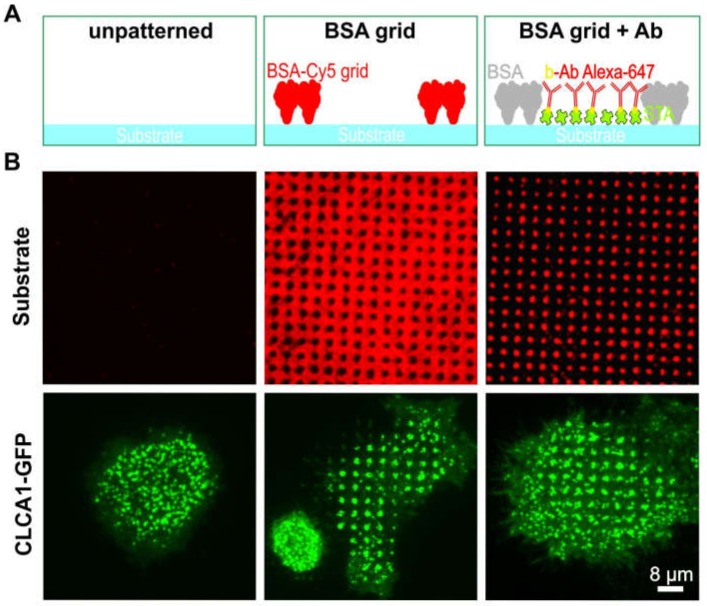
Effect of protein patterning on the distribution of clathrin in live cells. (**A**) Schematic illustration of the surface functionalization used in (**B**). (**B**) TIRF microscopy images of HeLa cells transiently expressing CLCA1-GFP. Left panel: plane glass substrate, middle panel: 1 µm BSA-Cy5 grid not filled with antibody, and right panel: 1 µm BSA grid filled with Alexa-647 labeled antibody (biotinylated). Abbreviations: b-Ab, biotinylated antibody; BSA, bovine serum albumin; and STA, streptavidin. Schematic illustrations are not drawn to scale.

We initially investigated the effect of BSA patterns on the distribution of clathrin in live cells (Figure 2). To this end, un-patterned surfaces (left column in Figure 2A,B) were compared with surfaces bearing micron-scale BSA grids (middle column) and anti-EGFR antibody spots (right column). HeLa cells transiently expressing GFP-labeled clathrin light-chain A1 (CLCA1-GFP) were allowed to adhere and grow for at least 4 h on the above-described surfaces. As expected, when the cells were grown on an unmodified glass substrate (Figure 2A,B, left), CLCA1-GFP was evenly clustered in the cell membrane. However, cells grown on the BSA-patterned substrate clearly revealed the presence of clathrin spots. Even the micron-scale BSA grid was sufficient to induce the accumulation of clathrin in the empty spaces between the grid (middle panel in Figure 2A,B). The observed patterns were similar to those of HeLa cells grown on spots bearing the anti-EGFR antibody in the spaces between the grid (Figure 2A,B, right panel), which confirms the observation in the previous study on the nature of CCP formation [12]. The live cell CLCA1-GFP patterning experiment exhibited large cell-to-cell and day-to-day variations, which results in 5–50% CLCA1-GFP pattern-positive cells.

**Figure 3:**
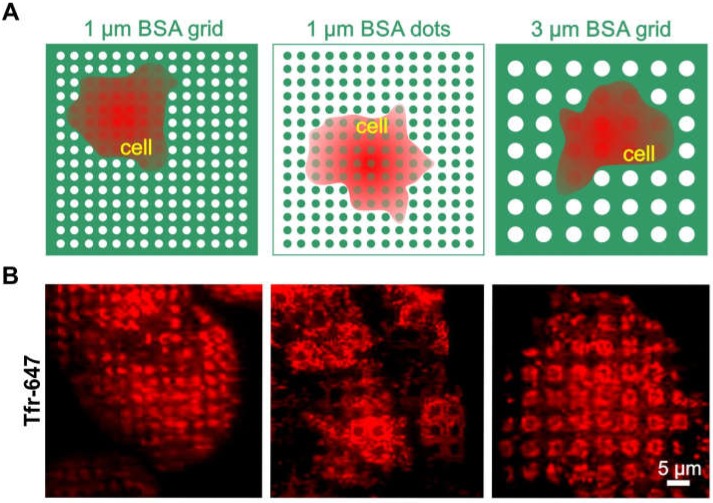
Clathrin distribution on different BSA patterns. (**A**) Schematic illustration of the surface functionalization used in (**B**). (**B**) TIRF microscopy images of HeLa cells grown on various surface patterns and stained for clathrin structures with Tfr-647 (red). Substrate functionalization is as follows: left—1 µm printed BSA grid, middle—1 µm printed BSA dots, and right—3 µm printed BSA grid. Schematic illustrations are not drawn to scale.

To further investigate this issue, we wanted to determine whether clathrin patterning is possibly independent of the pattern size and shape (Figure 3). For this purpose, HeLa cells were grown on 1 or 3 µm BSA grids and dots (Figure 3A), respectively, and were stained with Alexa-647 conjugated transferrin (Tfr-647), which is a standard marker for CCPs [31]. As shown in Figure 3B (left image), we observed clear clathrin patterns when cells were grown on the micron-scale BSA grid without any further functionalized modification. The results, therefore, confirmed the observations shown in the middle panel of Figure 2B. A comparable accumulation of clathrin in micropatterns was also detected in cells grown on a BSA grid with a 3-µm pattern size (Figure 3, right image). To prove the direct impact of substrate functionalization on clathrin distribution, an inverted stamp of the 1-µm grid layout (as shown in Figure 3A, left image) was used to print BSA dots instead of a grid structure. As expected, we also observed inverted patterning of the clathrin in cells grown on the dot substrate. Therefore, all data consistently showed that frequently used pattern shapes and sizes for µCP might be sufficient to directly affect clathrin rearrangement.

The above-described results obtained with micro-structured surfaces should be considered in light of previous studies in which patterned nanostructures were observed to directly affect CME via nanoscale bending and inward budding of the plasma membrane [32]. Although such nanoscale topographies are not completely comparable with the micron-scale topographies used, the void spaces between the raised spots could lead to a disturbance in the membrane architecture, which, in turn, would cause an accumulation of clathrin. We observed similar effects for clathrin distribution, as clathrin was preferably located in the rim structures of the patterns (Figure 3B, right image). This finding is in good agreement with a previously published study, indicating that both clathrin and dynamin2 showed strong preferences for the highly curved ends in cells grown on 3D nanostructures [32]. This further substantiates our hypothesis. Another explanatory approach is derived from reports that micropatterned surfaces spatially rearrange actin, which plays a variable role in CME [14,33,34]. Furthermore, it could also be possible that unspecific binding of serum components, which are present in the growth media, might occur within the bait-enriched areas. This could trigger receptor activation processes and subsequent formation of CCPs. Serum-free cell cultivation could be a possible means to investigate this question. In the past, we had made great effort to perform serum-free cell cultivation, e.g., by copatterning of adhesion molecules such as collagen, fibronectin, etc., since serum starving is frequently desired to bring cells into a basal state before analyzing signal transduction processes. However, with such approaches, we have not succeeded in creating a homogeneous cell-substrate interface, which is an indispensable requirement for quantitative TIRF microscopy. Although the exact mechanism of the observed clathrin patterns is not known and may vary depending on the cell type and substrate functionalization, our findings clearly show that this effect must be considered as a possibility when quantitatively investigating PPIs.

### 3.3. Formation of Clathrin Hotspots Leads to Unspecific Bait and Prey Copatterning

**Figure 4:**
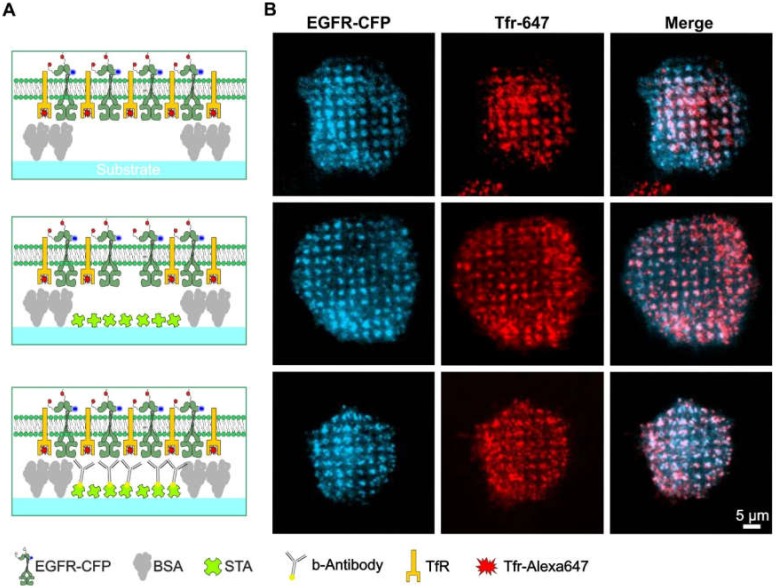
Bait molecules are rearranged irrespective of the presence of specific anti-bait molecules. (**A**) Schematic drawings of the different degrees of substrate functionalization are presented to the cells. (**B**) TIRF microscopy images of the corresponding HeLa cells are transiently expressing EGFR-CFP as the bait protein. Clathrin structures were visualized using Tfr-Alexa-647 staining (25 µg/mL for 10 min). Cells were stimulated with 170 nM EGF for 10 min prior to imaging. Abbreviations: b-antibody, biotinylated antibody; BSA, bovine serum albumin; STA, streptavidin; TfR, transferrin receptor; and Tfr, transferrin. Illustrations are drawn not to scale.

Since partial clathrin patterning leads to the formation of internalization hotspots, which can cause unspecific accumulation of different proteins in these assemblies, it is difficult to discriminate membrane-anchored bait proteins that are specifically enriched because of anti-bait proteins, such as antibodies and ligands. This problem is clearly illustrated in Figure 4. HeLa cells were transiently transfected with cyan fluorescent protein-labeled EGFR (EGFR-CFP) and grown on patterned substrates with increasing levels of substrate functionalization. Figure 4A depicts schematic illustrations of the substrate-cell interface with variations in substrate functionalization. In the upper panel, the substrate contained only a printed 1-µm BSA grid without further functionalization, whereas the middle panel shows the pattern that was also filled with streptavidin. Further functionalization of this pattern with biotinylated anti-EGFR antibody is shown in the bottom panel. As indicated in Figure 4B, stimulation of adhered HeLa cells with soluble EGF clearly revealed EGFR micropatterns to a comparable extent for all three degrees of substrate functionalization. These results show that the micron-scale BSA grid not only leads to clathrin patterning but also to unspecific EGFR copatterning (Figure 4, upper and middle panel). Strikingly, a comparable extent of EGFR copatterning was detected on substrates functionalized with anti-EGFR antibodies (Figure 4, bottom panel). However, the effects of the nonspecific protein patterning, i.e., cells grown on substrates without anti-bait antibodies, showed large fluctuations in pattern-positive cells.

Based on these findings, it is evident that interpreting a distinctive prey molecule copatterning is challenging, as the intended bait proteins might be enriched in the micropatterns due to specific anti-bait capture molecules. Additionally, other molecules, such as (endogenously expressed) receptors, which are constantly internalized via CME and potentially interact with the prey molecule of interest, can be enriched. Since such effects cannot be clearly resolved with “snapshot” mapping of individual cells (as shown in Figure 4B), it is necessary to quantify the results of the unspecific and specific patterning by statistical analysis.

**Figure 5:**
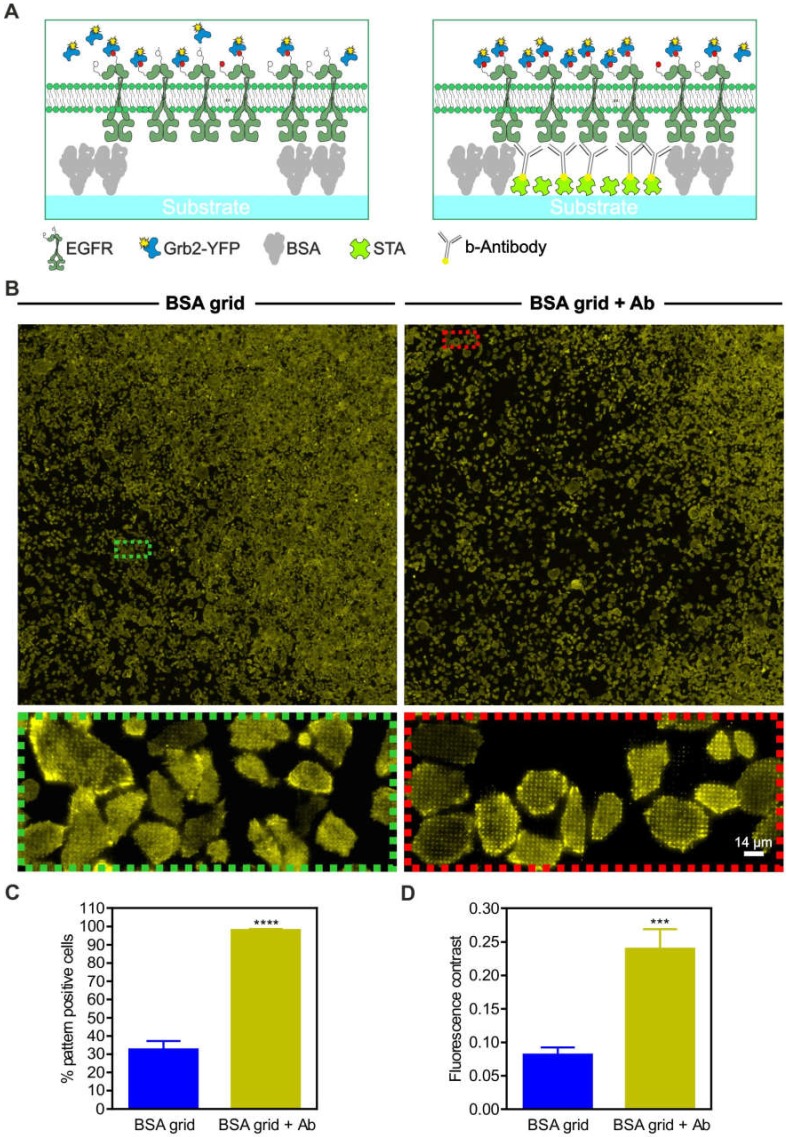
Specificity of prey protein copatterning. (**A**) Schematic illustrations of variations in the substrate-cell interface. (**B**) HeLa cells stably expressing Grb2-YFP were grown for at least 3–4 h on unmodified 1-µm BSA grids (left) or on completely functionalized anti-EGFR antibody-coated surfaces (right). Ten minutes after EGF stimulation (170 nM), large-area surface scans were captured to deliver a representative snapshot of the Grb2-YFP distribution. Insets show enlarged areas of the overall scans. (**C**) Quantitation of the number of pattern-positive cells. (**D**) Quantitation of fluorescence contrast. Error bars are based on the mean ± SE of more than 300 cells. *** *p* < 0.05 and **** *p* < 0.001 for comparison of different levels of substrate functionalization. Graphical illustrations are not drawn to scale.

For further evaluation of the unspecific prey co-recruitment, we performed large-area substrate scans of adhered live HeLa cells stably expressing Grb2-YFP (Figure 5). The cells were grown on 1-µm BSA grids with or without anti-EGFR antibody functionalization (Figure 5A,B). We found that approximately 30% of the cells grown on the BSA grid without antibody functionalization showed at least slight copatterning of Grb2-YFP (Figure 5C). In contrast, the cells grown on anti-EGFR antibody-patterned surfaces revealed a significantly increased extent of Grb2-YFP copatterning (~100%, *p* < 0.0001) with respect to the number of pattern-positive cells. This finding shows that the specific accumulation of EGFR in micropatterns and the Grb2 interaction occurs on nonfunctionalized patterns, but both were less pronounced (mean fluorescence contrast 0.08 ± 0.01) than they were in the presence of the specific capture protein (mean fluorescence contrast 0.24 ± 0.03, *p* < 0.001) (Figure 5D).

These results indicate that comprehensive controls are necessary for the accurate quantitative analysis of the specific EGFR-Grb2 interaction. Furthermore, the presence of other receptors should be taken into consideration, particularly those reported to interact with Grb2 and are internalized via CME, such as the ephrin type-A1 receptor (EphA1) [35,36], c-Met receptor [37,38], platelet-derived growth factor receptor β (PDGFRβ) [39,40], fibroblast growth factor receptor 2 (FGFR2) [41,42], and β_2_-adrenergic receptors (β_2_Ars) [43,44]. These receptors might potentially contribute to copatterning and, thus, impair the quantitative analysis of the bait-prey interaction of interest.

### 3.4. Towards more Reliable Live Cell Micropatterning Experiments

The above-described effects are not limited to the arrangement of membrane-anchored bait and cytosolic prey molecules. In cases where both interaction partners (bait as well as prey) are membrane-bound, the likelihood of misinterpretation and detection of false-positive copatterning may be even higher since both interaction partners can be directly affected by CME. This potential problem might be of particular concern in studies of cellular signaling based on receptor heterodimerization and oligomerization and receptor transactivation studies. Due to these circumstances, a detailed control and critical analysis of the putative bait-prey interactions studied on microstructure substrates are essential.

To prevent the misinterpretation and quantitation of false-positive copatterning events, the following experimental controls and suggestions should be considered.

1) From a general point of view, each bait/prey interaction pair under investigation requires independent validation with respect to the reported effects. For example, prior to experiments, preferred desensitization routes of the receptors under investigation should be elaborated and the likelihood of unspecific bait/prey co-recruitment on patterned surfaces with different substrate functionalization should be determined (e.g., as shown in Figure 4).
2) The formation of internalization hotspots within the micropatterned areas should be tested. This strategy might not be limited to CME since other pathways of desensitization, such as caveolin-dependent or clathrin/caveolin-independent endocytosis, may as well have an effect on the PPIs [45]. While it could be shown that non-CME endocytic proteins, such as caveolin1, are not enriched on patterned substrates [32], careful analysis through the use of control markers (e.g., fluorescent cavin fusion proteins [46], bulk phase markers such as Lucifer yellow [47], or specific monovalent fluorescent ligand conjugates [48]) is mandatory.
3) In the case of solely membrane-bound interaction partners, the bait and prey surface proteins should be accordingly exchanged to prove co-patterning under different bait/prey conditions. As an example of such a control experiment, we describe a thought experiment based on the putative interaction between the EGFR and MHC (major histocompatibility complex) class I molecules [49]. In the first set of experiments, the EGFR serves as bait, and the MHC class I molecule is used as the prey protein, while copatterning is analyzed. Subsequently, the MHC class I molecule is used as the bait, and the EGFR serves as the prey. Again, copatterning can be analyzed, and putative differences are evaluated with respect to the influence of CME. This is of special importance if one of the possible interaction partners is not internalized via CME, such as MHC class I molecules [50], as this could directly affect the conclusions on whether the copatterning was influenced by clathrin hotspots.
4) Mutant variants of the bait as well as the prey proteins should be tested in control experiments. For instance, the mutation of important interaction domains and of tyrosine residues that are phosphorylated can be used for the investigation and independent confirmation of specific bait/prey copatterning. Likewise, the use of appropriate agonists/antagonists can help determine the specificity of the detected protein patterning. For example, known inhibitors of receptors or PPIs can be used to confirm and possibly even quantify the specificity of the interaction of interest.
5) In addition to biological-based considerations, we also suggest pursuing different technological strategies. First, implementation of large-area surface scans (as shown in Figure 5) in combination with single-cell analysis allows for unbiased identification of different cell populations that can reveal deviations in the quantitative parameters. Second, when using quantitative TIRF microscopy, a homogeneous substrate-cell membrane interface is a prerequisite to avoid false positives. This can be achieved, for instance, by imaging the cell membrane in TIR using specific stains for lipophilic membranes such as DiD oil (1,1′-dioctadecyl-3,3,3′,3′-tetramethylindodicarbocyanine perchlorate) or respective membrane-anchored fluorescent fusion proteins [51].

Lastly, different substrate functionalization strategies can be elaborated to identify (as done here) and potentially minimize the formation of clathrin hotspots within the patterned areas in the cell. Since this technical approach certainly involves the highest experimental effort, a modular functionalization strategy is desirable. Ideally, patterning technology should also enable spatial resolution on the nanometer length scale to allow for the study of nanoscale effects, particularly in the early stages of cell signaling [52,53]. DNA surface technology offers these possibilities, since DNA oligonucleotides can be used as programmable highly modular linker systems, which can even be designed to provide nanostructure scaffolds for ligand attachment. In this regard, we have recently developed multi-scale origami structures as interfaces for cells (MOSAIC) [24]. Using MOSAIC, micropatterned arrays of DNA capture oligonucleotides were prepared by polymer pen lithography (PPL) and, subsequently, decorated with DNA origami nanostructures that presented variable numbers of EGF ligands in a highly defined spatial arrangement on a nanometer length scale. Upon testing these surfaces for the activation of the EGFR in living MCF7 cells, we demonstrated that the nanoscale architecture of this MOSAIC system specifically influenced EGFR activation [24]. Based on this promising preliminary study, we wanted to determine whether the microplate technology described above is also compatible with DNA linker systems and MOSAIC.

### 3.5. Implementation of DNA Linker Systems and DNA Origami Nanostructures for Interaction Analysis in Live Cells

**Figure 6:**
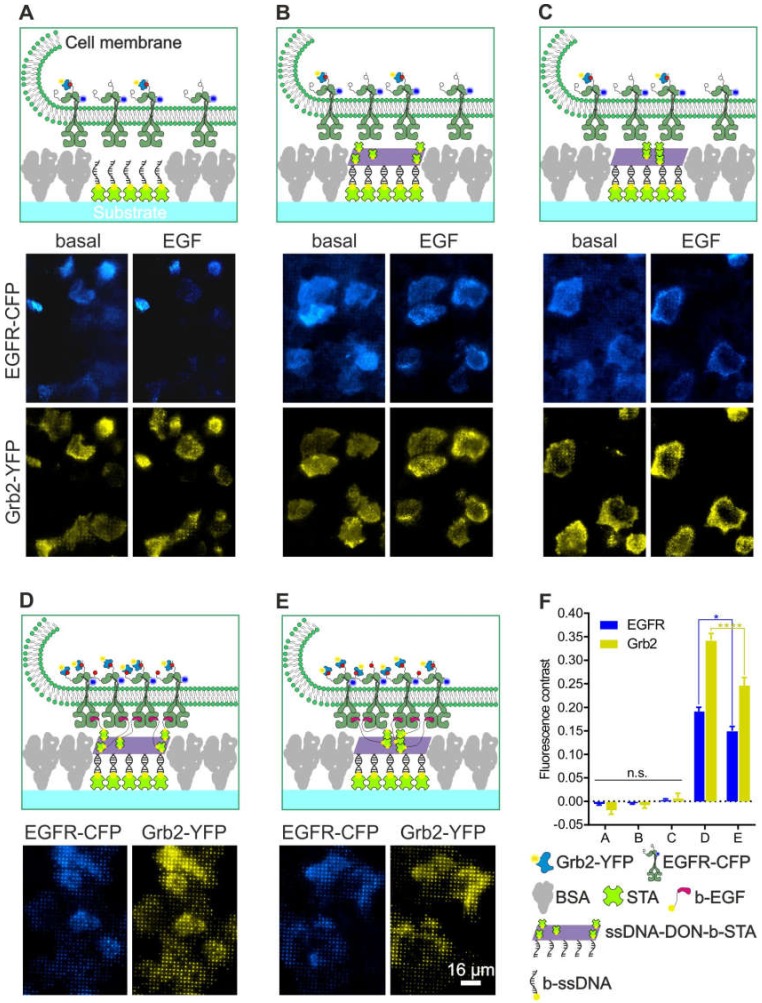
Impact of the degree of surface functionalization on the specificity of bait (EGFR) and prey (Grb2) enrichment using a DNA origami approach. HeLa cells transiently co-expressing EGFR-CFP and Grb2-YFP were grown on 1-µm BSA grids consisting of different surface chemistry as in (**A**) single-stranded oligonucleotides, (**B**) hybridized DONs decorated with the 5far arrangement of STA, (**C**) chemistry as in B but with the 5close DON arrangement, (**D**) chemistry as in B but additionally modified with biotinylated EGF to mediate specific EGFR capturing and activation, (**E**) chemistry as shown in D but with 5close DON. Where applicable, the cells were stimulated with EGF (170 nM) for at least 5-10 min prior to imaging. (**F**) Quantitation of EGFR-CFP and Grb2-YFP fluorescence contrast of the cells grown on the substrates shown in A-E. Error bars are based on the mean ± SE of more than 150 cells. * *p* < 0.05 and **** *p* < 0.001 for comparisons of 5far and 5close DONs. Quantitation is based on Figure S2, Figure S3, Figure S4, Figure S5 and Figure S6. Abbreviations: BSA, bovine serum albumin; b-EGF, biotinylated EGF; b-ssDNA, biotinylated single-stranded DNA; STA, streptavidin; and ssDNA-DON-b-STA, single-stranded DON with biotin and streptavidin. Graphical illustrations are not drawn to scale.

To experimentally simplify and increase the throughput of the MOSAIC technology, we adopted a DNA-based method using the micro-structured 96-well plate produced by µCP to investigate the specificity of bait/prey enrichment as a function of complete surface functionalization (Figure 6). For this purpose, streptavidin was first patterned in between the BSA grids and, subsequently, modified with biotinylated single-stranded oligonucleotides. After saturation of the remaining biotin-binding sites with free biotin, the oligonucleotide-modified surface then served to capture different DNA origami nanostructures (DONs) by means of specific Watson-Crick hybridization. The DONs were decorated with five streptavidin molecules arranged in either a close or a far configuration (Figure S1). HeLa cells transiently expressing EGFR-CFP and Grb2-YFP were used for the investigation of the various surfaces. When cells were grown on surfaces presenting only single-stranded oligonucleotides, few single cells showing some EGFR and Gbr2 corecruitment to the oligonucleotide pattern were visible in a small number of snapshots (Figure 6A). Similar homogeneous bait/prey distribution was found in cells grown on surfaces functionalized with either 5close (Figure 6B) or 5far (Figure 6C) DONs that carried only “naked” streptavidin on their surface.

In marked contrast to these results, the degree of specific EGFR and Grb2 copatterning was significantly enhanced when biotinylated EGF patterns were presented to the cells via DONs (Figure 6D,E). To supplement the snapshots shown in Figure 6, a surface scanning approach (such as that shown in Figure 5) was conducted to allow for objective and quantitative evaluation of specific/unspecific EGFR-CFP/Grb2-YFP patterning. For this purpose, 200–300 captured images were automatically assembled into a single high-resolution image for each condition (Figures S2–S6). Based on this large-area imaging approach, the fraction of cells showing bait/prey copatterning, as well as the mean fluorescence contrast based on different degrees of substrate functionalization, was quantitated (Figure 6F). We found a less than 1% of the cell population showing unspecific bait/prey copatterning after EGF stimulation when grown on substrates without specific anti-bait protein (as shown in Figure 6A–C). This resulted in a mean fluorescence contrast of ~0, which indicates homogeneous bait/prey distribution in the cell membrane. In contrast, 90–100% of the cells exhibited significant bait/prey copatterning when EGF patterns were presented to the cells (Figure 6D,E) with a mean fluorescence contrast of 0.15–0.19 (EGFR) and 0.25–0.34 (Grb2). In agreement with our previous study on MCF7 cells [24], both the recruitment of EGFR (*p* < 0.05) and Grb2 (*p* < 0.001) was significantly higher in the case of the 5far configuration than it was in the 5close configuration of the EGF ligands presented on the DONs. In order to further demonstrate the specificity of the adapted MOSAIC approach, we used a previously reported Grb2 mutant (mSH2-Grb2) containing a R86K mutation (arginine 86 to lysine) in the SH2 domain. This mutation was shown to disrupt the SH2-dependent membrane recruitment and EGFR interaction [28]. We found a substantially reduced Grb2 copatterning in Hela cells transiently transfected with mSH2-Grb2-YFP as compared to cells expressing wildtype Grb2-YFP (Figure S7).

Taken together, these results are very important for several reasons. First, the use of DNA linker systems is associated with a much lower degree of clathrin accumulation. The reasons for this are unclear but could be related to the fact that DNA, as a charged and highly hydrated molecule, is more effective in filling the voids between the BSA grids than were either the naked surface or streptavidin coatings (as used in the examples shown in Figure 2, Figure 3, Figure 4 and Figure 5). On the other hand, the results clearly confirm the transferability of the MOSAIC technology, both in terms of compatibility with alternative surface functionalization methods and the cells used. Overall, this initial assessment confirms the observations previously made indicating that DNA-based protein micropatterning has clear advantages over simple protein patterning [54,55].

## 4. Conclusions

Protein micropatterning in combination with single-cell analysis has evolved into a powerful tool for basic and advanced research, as proteins and related interactions, as well as signal transduction processes, can be investigated in a native protein and lipid environment and in a live cell context. However, to tap the full potential of the introduced methodology, thorough controls and deliberate experimental design are necessary. In summary, we presented strong evidence that protein patterning at micron-scale distances can induce the formation of clathrin hotspots within patterned areas. We discussed that this phenomenon might directly affect the specificity of bait/prey co-recruitment and the respective quantitative analyses. Therefore, novel strategies that reduce the described phenomenon while simultaneously enabling specific bait patterning are highly desired. In this regard, DNA-based surface patterning seems to favor the formation of clathrin hotspots to a lesser extent. We could show that the sophisticated surface functionalization making use of the MOSAIC methodology in combination with PPL can be easily transferred to preassembled substrate structures (e.g., generated by µCP). The resulting surface-patterning approach combines the strength of both strategies. On the one hand, µCP is a simple and robust procedure for substrate patterning that also allows for large substrate functionalization with high reproducibility. On the other hand, the MOSAIC technology enables specific and highly defined spatial arrangements of anti-bait proteins on a nanometer length scale with reduced formation of clathrin hotspots. We intend to use this highly modular protein patterning approach to quantitatively investigate early stages of receptor-mediated signal transduction processes in the future.

## Supplementary Materials

The following are available online at https://www.mdpi.com/2218-273X/10/4/540/s1. Figure S1: Design and analysis of DNA origami nanostructures. Figure S2: Impact of the degree of surface functionalization on the specificity of the bait (EGFR) and prey (Grb2) enrichment using a DNA origami approach. Figures S3–S7: Impact of the degree of surface functionalization on the specificity of the bait (EGFR) and prey (Grb2) enrichment using a DNA origami approach.
