# Supplementary material for: Highly Modular Protein Micropatterning Sheds Light on the Role of Clathrin-Mediated Endocytosis for the Quantitative Analysis of Protein-Protein Interactions in Live Cells
Source: Biomolecules. 2020 Apr 2;10(4):540. doi: 10.3390/biom10040540 (PMC7225972; doi:10.3390/biom10040540)
Supplement: Supplementary file 1 [file biomolecules-10-00540-s001.pdf]

Supplementary Figures

Highly Modular Protein Micropatterning Sheds Light on the Role of Clathrin-Mediated Endocytosis for the Quantitative Analysis of Protein-Protein Interactions in Live Cells

Peter Lanzerstorfer [1,*], Ulrike Müller [1], Klavdiya Gordiyenko [2], Julian Weghuber [1,*] and Christof M. Niemeyer [2,*]

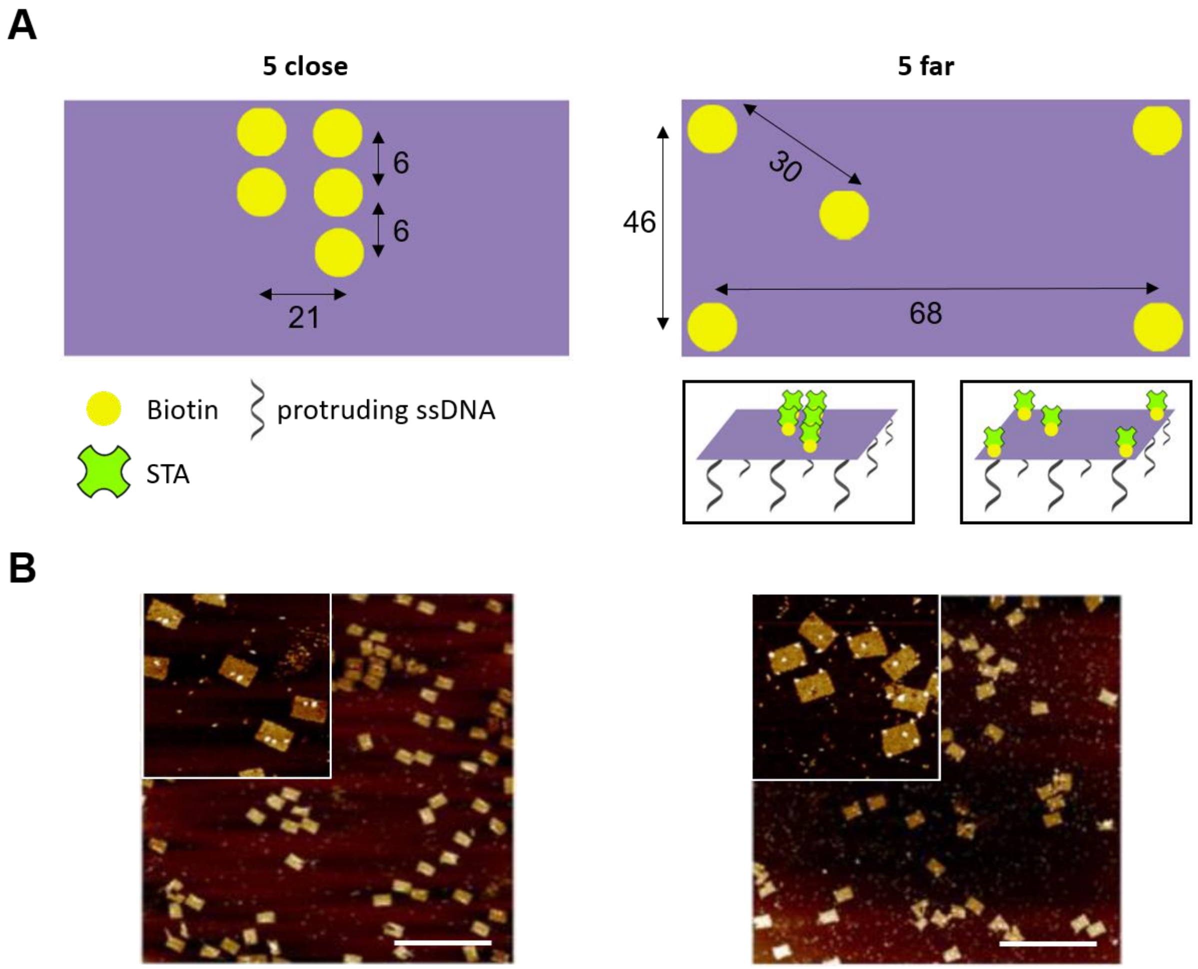

Figure S1. Design and analysis of DNA origami nanostructures. (A) Schematic illustrations of the two different DON conformations "5close" (left) and "5far" (right). The distances between the biotin groups are indicated in nanometers. The insets illustrate the 3D orientation of the DONs bearing STA and single-stranded protruding oligomers for hybridization with surface-bound capture oligonucleotides on the upper and lower sides, respectively. Illustrations are not drawn to scale. (B) Representative AFM images of the DONs decorated with streptavidin. Scale bar: 500 nm.

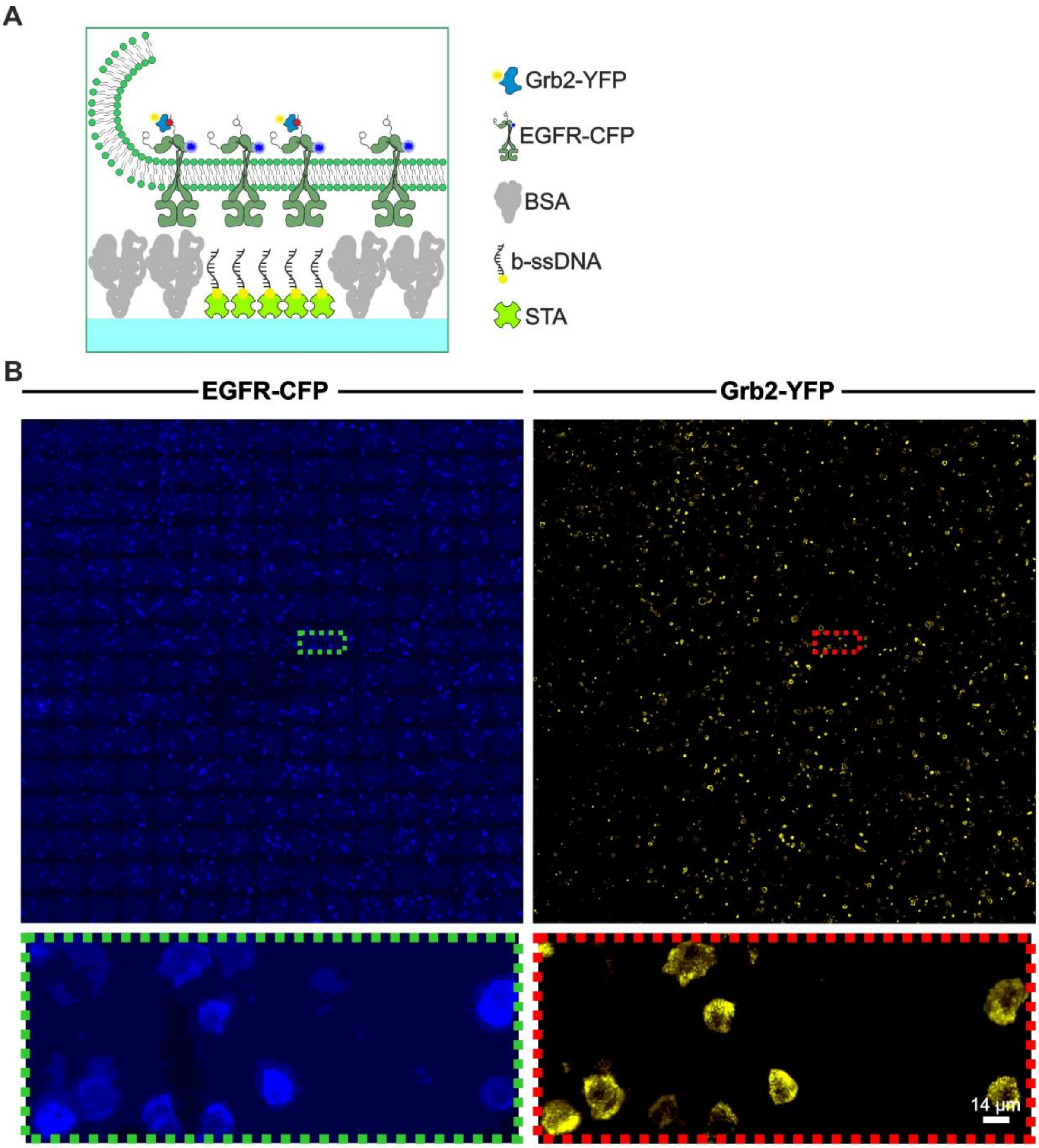

Figure S2. Impact of the degree of surface functionalization on the specificity of the bait (EGFR) and prey (Grb2) enrichment using a DNA origami approach. (A) Schematic illustration of the substrate-cell interface. (B) HeLa cells transiently coexpressing EGFR-CFP and Grb2-YFP were grown on 1 µm BSA grids consisting of single-stranded oligonucleotides as shown in (A). Ten minutes after EGF stimulation (170 nM), large-area surface scans were captured to provide a representative snapshot of the bait and prey distribution. Insets show enlarged areas of the overall scans. Graphical illustrations are not drawn to scale. Abbreviations: BSA, bovine serum albumin; b-ssDNA, biotinylated single-stranded DNA; and STA, streptavidin.

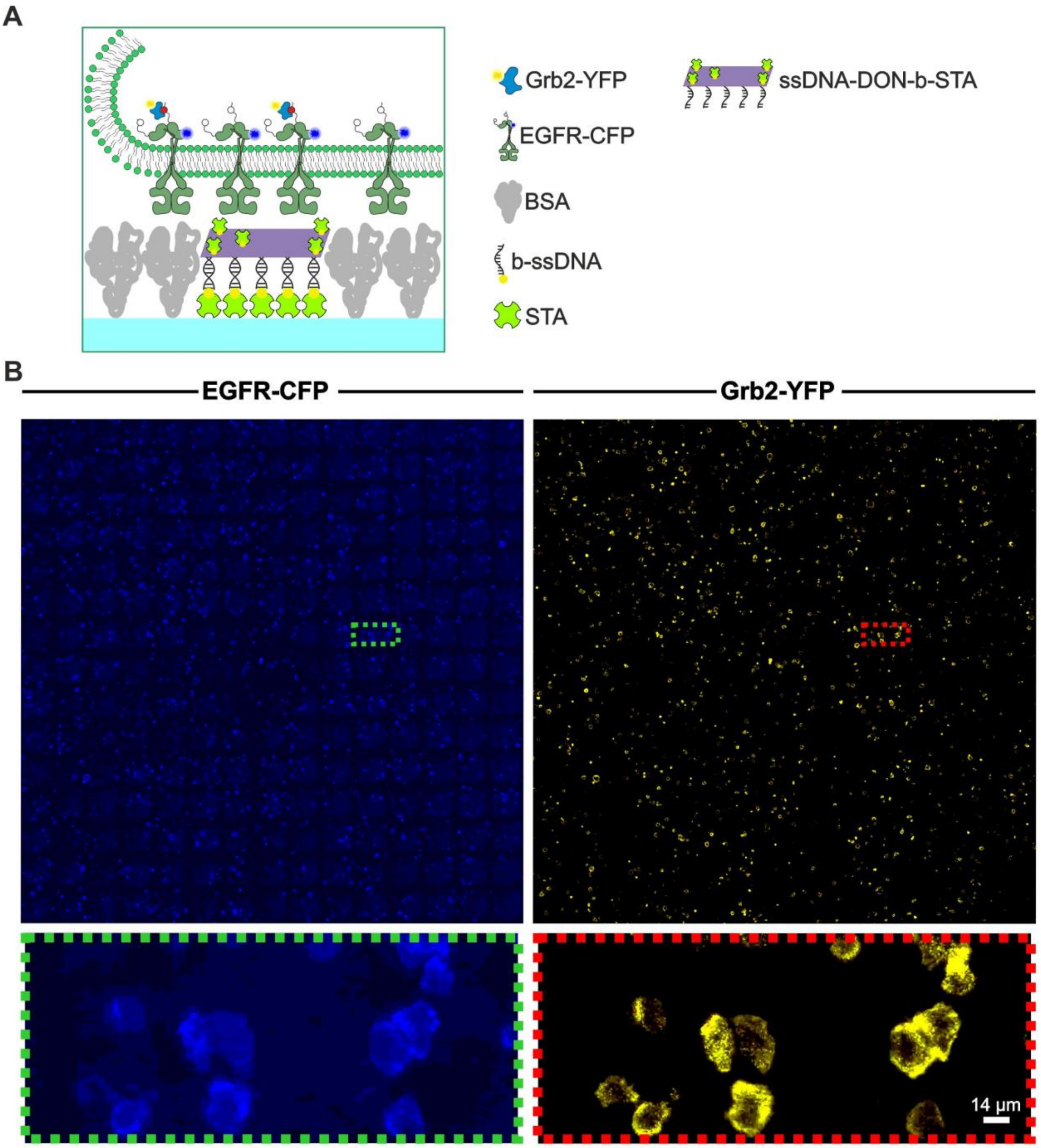

Figure S3. Impact of the degree of surface functionalization on the specificity of the bait (EGFR) and prey (Grb2) enrichment using a DNA origami approach. (A) Schematic illustration of the substrate-cell interface. (B) HeLa cells transiently coexpressing EGFR-CFP and Grb2-YFP were grown on 1 µm BSA grids consisting of hybridized DON decorations in a 5far arrangement of STA, as shown in (A). Ten minutes after EGF stimulation (170 nM), large-area surface scans were captured to provide a representative snapshot of the bait and prey distribution. Insets show enlarged areas of the overall scans. Graphical illustrations are not drawn to scale. Abbreviations: BSA, bovine serum albumin; b-ssDNA, biotinylated single-stranded DNA; STA, streptavidin; and ssDNA-DON-b-STA, single-stranded DON with biotin and streptavidin.

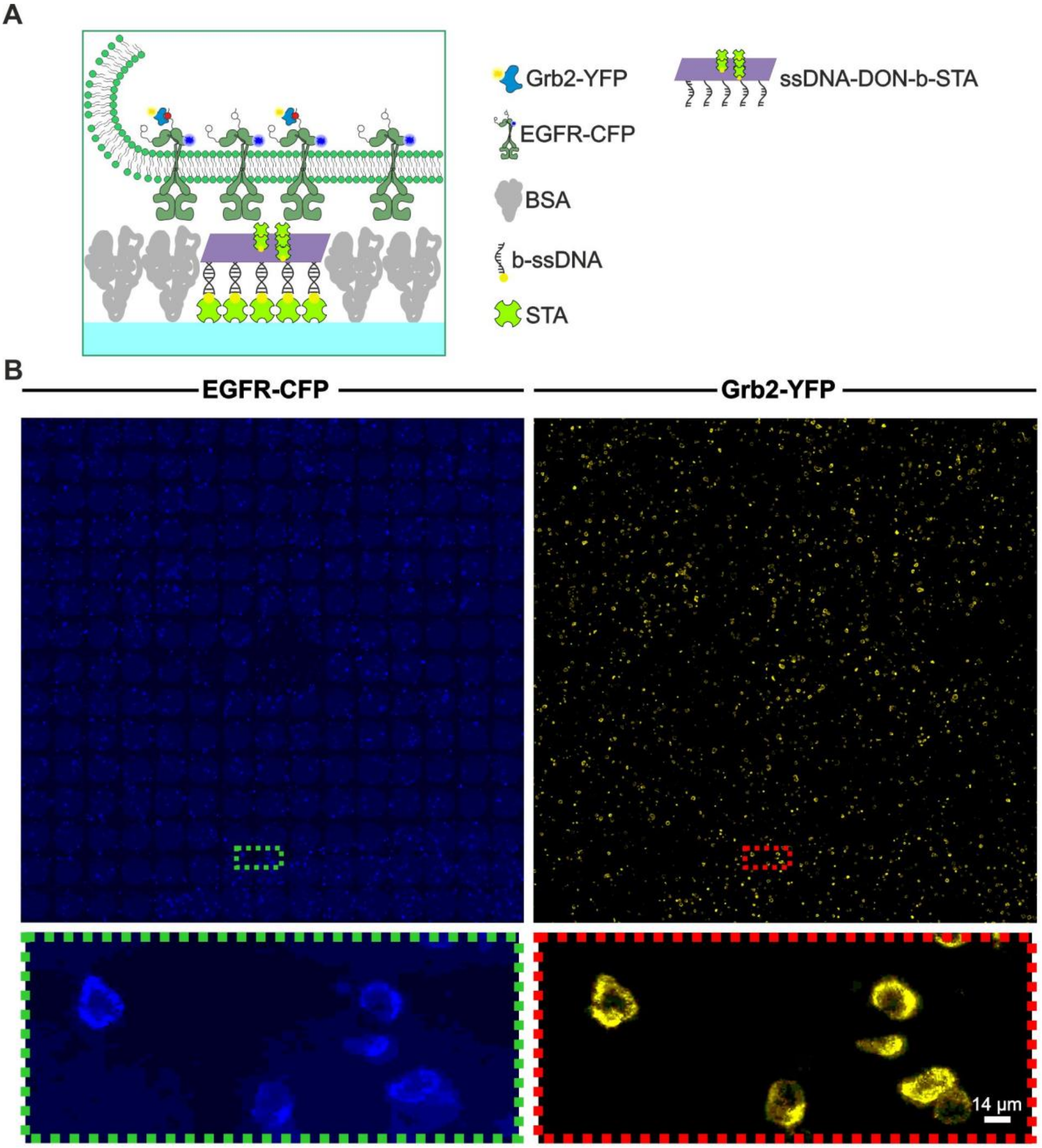

Figure S4. Impact of the degree of the surface functionalization on the specificity of the bait (EGFR) and prey (Grb2) enrichment using a DNA origami approach. (A) Schematic illustration of the substrate-cell interface. (B) HeLa cells transiently coexpressing EGFR-CFP and Grb2-YFP were grown on 1 µm BSA grids consisting of hybridized DON decorated with a 5close arrangement of STA, as shown in (A). Ten minutes after EGF stimulation (170 nM), large-area surface scans were captured to provide a representative snapshot of the bait and prey distribution. Insets show enlarged areas of the overall scans. Graphical illustrations are not drawn to scale. Abbreviations: BSA, bovine serum albumin; b-ssDNA, biotinylated single-stranded DNA; STA, streptavidin; and ssDNA-DON-b-STA, single-stranded DON with biotin and streptavidin.

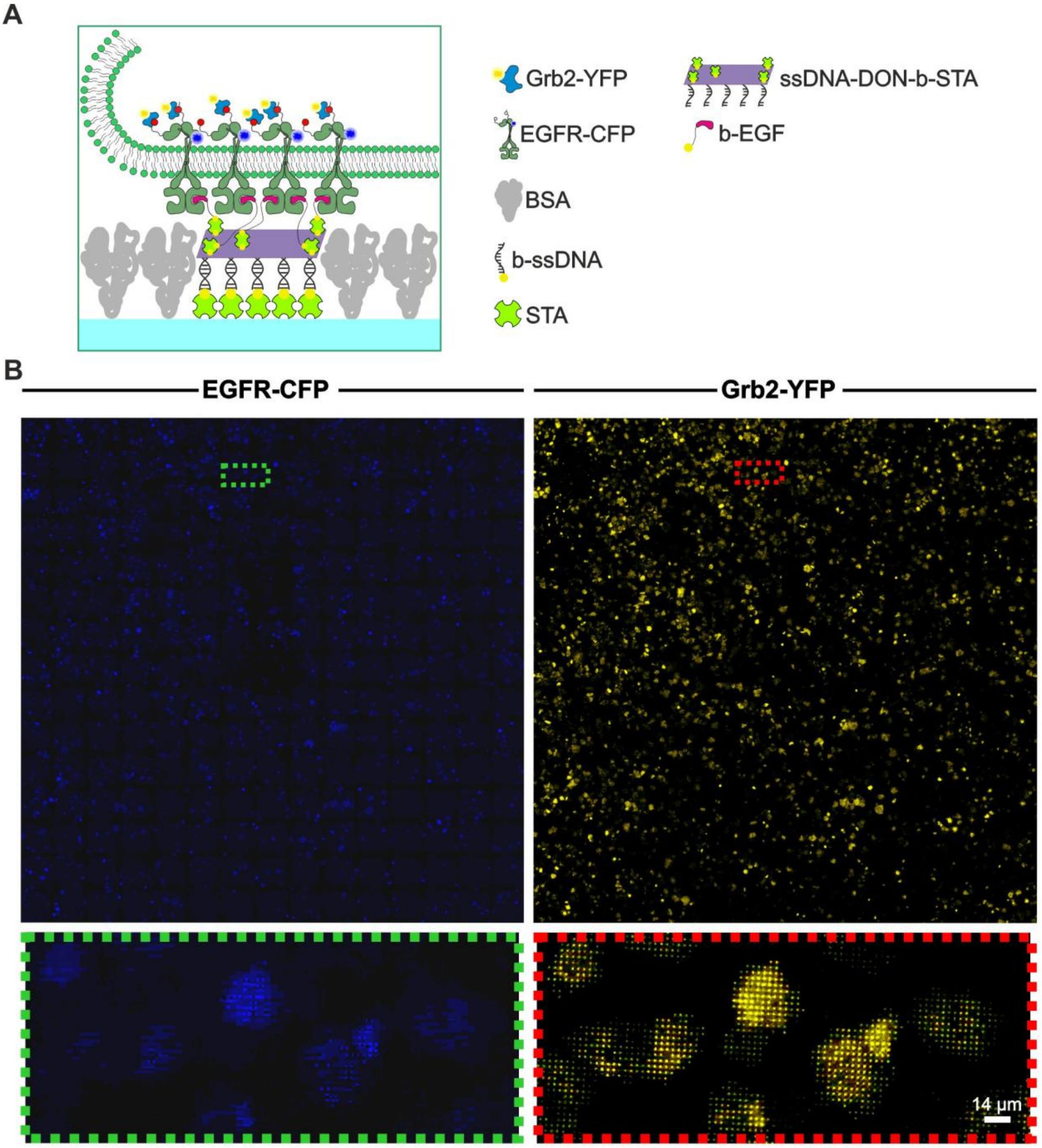

Figure S5. Impact of the degree of surface functionalization on the specificity of the bait (EGFR) and prey (Grb2) enrichment using a DNA origami approach. (A) Schematic illustration of the substrate-cell interface. (B) HeLa cells transiently coexpressing EGFR-CFP and Grb2-YFP were grown on 1 µm BSA grids consisting of hybridized DON decorated with a 5far arrangement of STA, as shown in (A), and additionally modified with biotinylated EGF to mediate specific EGFR capture and activation. Large-area surface scans were taken to obtain a representative overview of the bait and prey distribution. Insets show enlarged areas of the overall scans. Graphical illustrations are not drawn to scale. Abbreviations: BSA, bovine serum albumin; b-EGF, biotinylated EGF; b-ssDNA, biotinylated single-stranded DNA; STA, streptavidin; and ssDNA-DON-b-STA, single-stranded DON with biotin and streptavidin.

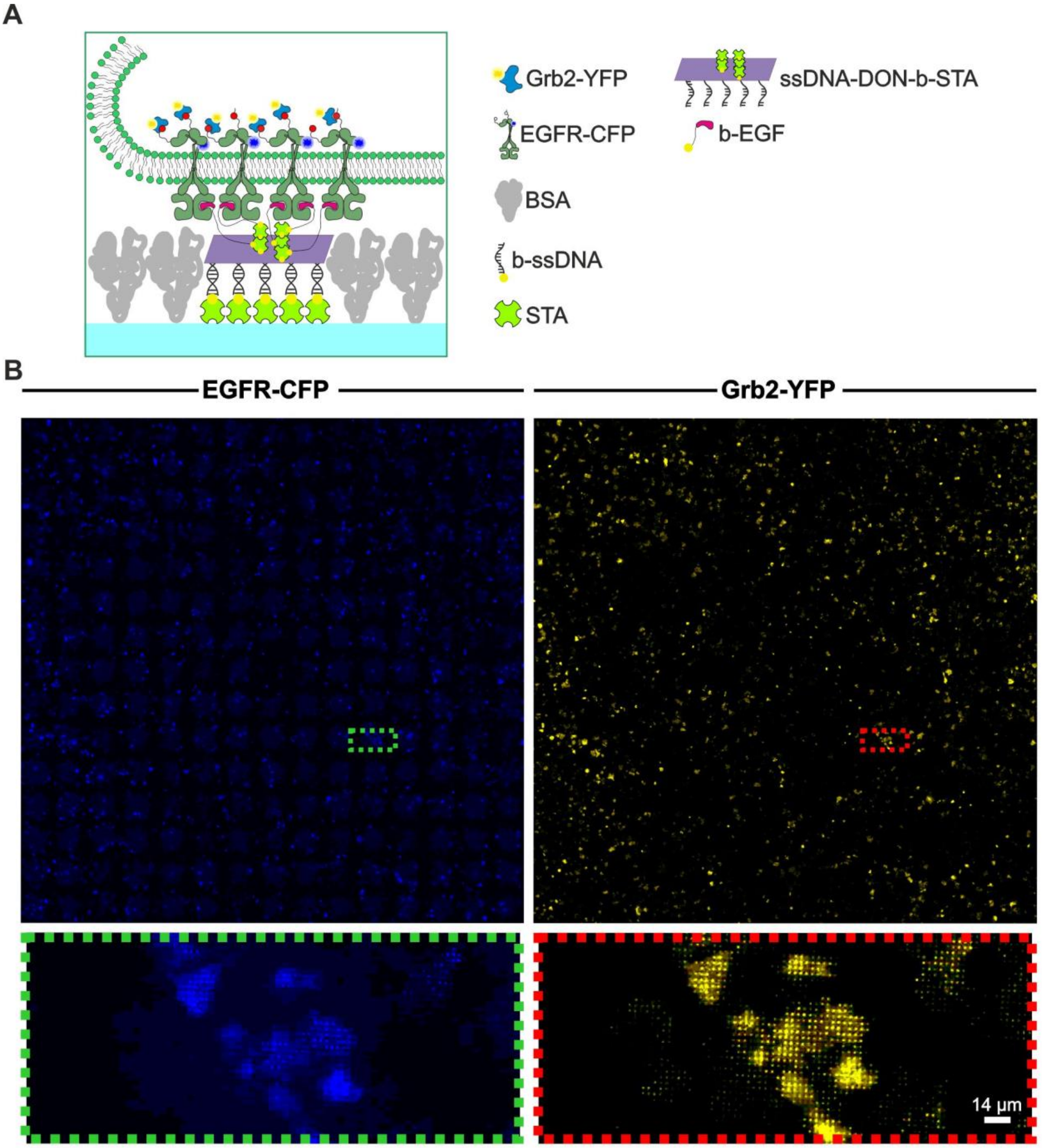

Figure S6. Impact of the degree of surface functionalization n the specificity of bait (EGFR) and prey (Grb2) enrichment using a DNA origami approach. (A) Schematic illustration of the substrate-cell interface. (B) HeLa cells transiently coexpressing EGFR-CFP and Grb2-YFP were grown on 1 µm BSA grids consisting of hybridized DON decorated with a 5close arrangement of STA, as shown in (A), and additionally modified with biotinylated EGF to mediate specific EGFR capture and activation. Large-area surface scans were taken to obtain a representative overview of the bait and prey distribution. Insets show enlarged areas of overall scans. Graphical illustrations are not drawn to scale. Abbreviations: BSA, bovine serum albumin; b-EGF, biotinylated EGF; b-ssDNA, biotinylated single-stranded DNA; STA, streptavidin; and ssDNA-DON-b-STA, single-stranded DON with biotin and streptavidin.

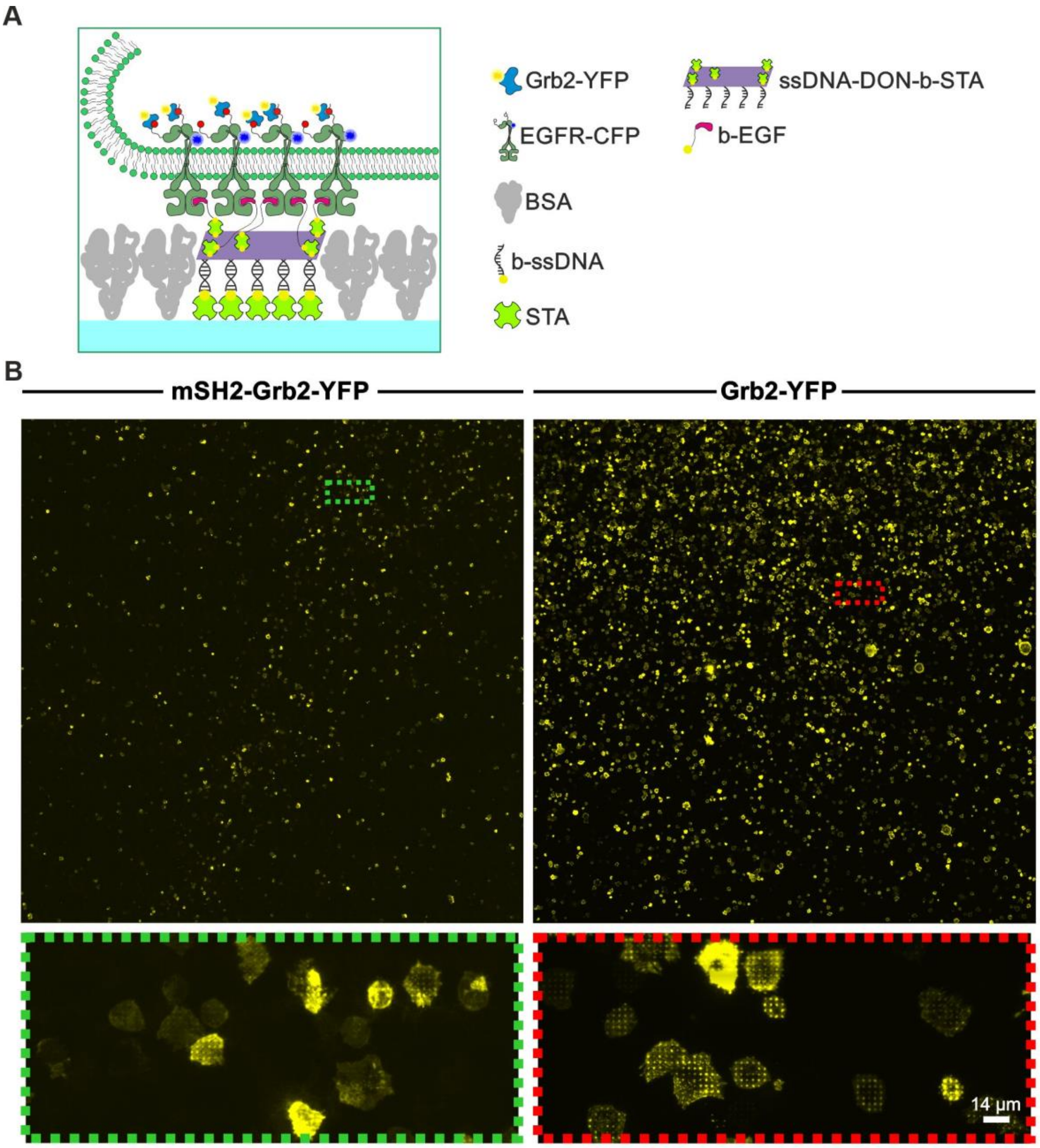

Figure S7. mSH2-Grb2 shows substantially reduced copatterning in comparison to wildtype Grb2. (A) Schematic illustration of the substrate-cell interface. (B) HeLa cells transiently expressing mSH2-Grb2-YFP (left) and Grb2-YFP (right) were grown on 1 µm BSA grids consisting of hybridized DON decorated with a 5far arrangement of STA-bEGF to mediate specific EGFR capture and activation. Large-area surface scans were taken to obtain a representative overview of the bait and prey distribution. Insets show enlarged areas of overall scans. Note that the EGF-dependent co-localization of the mSH2-Grb2 mutant with the EGFR micropatterns is significantly reduced (yellow spots in the large-area scans). However, due to the high sensitivity of TIRF microscopy, in some cells a slight co-recruitment can still be detected (see green framed insert). This observation suggests that this particular mutation in the SH2 domain does not completely inhibit EGFR-Grb2 interaction. Graphical illustrations are not drawn to scale. Abbreviations: BSA, bovine serum albumin; b-EGF, biotinylated EGF; b-ssDNA, biotinylated single-stranded DNA; STA, streptavidin; and ssDNA-DON-b-STA, single-stranded DON with biotin and streptavidin.
